# FEA Comparison of the Mechanical Behavior of Three Dental Crown Materials: Enamel, Ceramic, and Zirconia

**DOI:** 10.3390/ma17030673

**Published:** 2024-01-30

**Authors:** Mario Ceddia, Luciano Lamberti, Bartolomeo Trentadue

**Affiliations:** Dipartimento di Meccanica, Matematica e Management, Politecnico di Bari, 70125 Bari, Italy; m.ceddia@phd.poliba.it (M.C.); bartolomeo.trentadue@poliba.it (B.T.)

**Keywords:** dental stress analysis, finite element analysis, crown, dentin, crown materials, prosthetic dentistry

## Abstract

The restoration of endodontically treated teeth is one of the main challenges of restorative dentistry. The structure of the tooth is a complex assembly in which the materials that make it up, enamel and dentin, have very different mechanical behaviors. Therefore, finding alternative replacement materials for dental crowns in the area of restorative care isa highly significant challenge, since materials such as ceramic and zirconia have very different stress load resistance values. The aim of this study is to assess which material, either ceramic or zirconia, optimizes the behavior of a restored tooth under various typical clinical conditions and the masticatory load. A finite element analysis (FEA) framework is developed for this purpose. The 3D model of the restored tooth is input into the FEA software (Ansys Workbench R23)and meshed into tetrahedral elements. The presence of masticatory forces is considered: in particular, vertical, 45° inclined, and horizontal resultant forces of 280 N are applied on five contact points of the occlusal surface. The numerical results show that the maximum stress developed in the restored tooth including a ceramic crown and subject to axial load is about 39.381 MPa, which is rather close to the 62.32 MPa stress computed for the natural tooth; stresses of about 18 MPa are localized at the roots of both crown materials. In the case of the zirconia crown, the stresses are much higher than those in the ceramic crown, except for the 45° load direction, while, for the horizontal loads, the stress peak in the zirconia crown is almost three times as large as its counterpart in the ceramic crown (i.e., 163.24 MPa vs. 56.114 MPa, respectively). Therefore, the zirconia crown exhibits higher stresses than enamel and ceramic that could increase in the case of parafunctions, such as bruxism. The clinician’s choice between the two materials should be evaluated based on the patient’s medical condition.

## 1. Introduction

**Figure 1:**
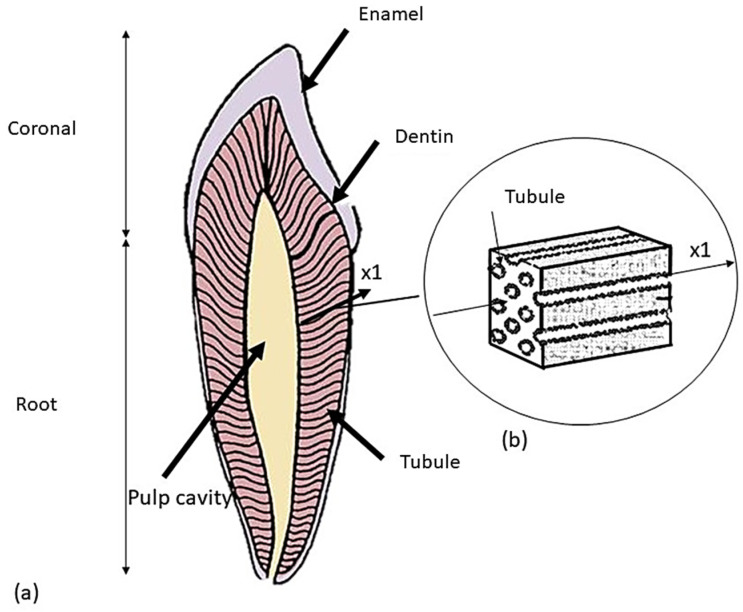
Schematic of a premolar tooth and the corresponding dentin microstructure: (**a**) tooth longitudinal section; (**b**) representation of the volume element extracted in the ×1 direction of the tubule.

Employing artificial crowns is a typical method in prosthetic dentistry for recreating the natural dental structure to solve problems, such as cavities and other structural injuries. Materials such as ceramics and metals have been very commonly used for prosthetic restoration, supported by natural teeth or implants [1,2]. Enamel makes up the natural tooth crown of the tooth, grinds food, and protects dentin, which acts as a force absorber during chewing. Dentin is a hard bone-like material that has an inner structure comprising a large number of tubules with variable diameter and spacing: this results in its anisotropic behavior. A study assessed the effect of various acids in cleaning the tooth surface, revealing that the use of polyacrylic acid is advantageous compared to other acids [3]. Some researchers [4] studied the influence of dentin tubules on mechanical characteristics. In particular, Kinney et al. [5] adopted a micromechanics-based approach to study the physical properties of dentin. Another approach followed in the literature was to take the transverse tubule of the dentin as a reference system and record the variation in mechanical properties along the tubule [6,7,8]. In particular, dentin was assessed to behave as a transversely isotropic material along the direction of the tubules (×1) (see Figure 1, taken from Ref. [8]).

In the late1990s, the achieved developments in the field of experimental techniques allowed the knowledge of dentin micromechanical behavior to be significantly improved [9]. For example, Wang et al. [10] used the intrinsic moiré structure to map strain distributions in the plane of the applied compressive load. It was found that dentin should be regarded as a non-homogeneous anisotropic material rather than homogeneous and isotropic. Kinney et al. [11] investigated the mechanical properties of dentin; in particular, the measured values of the Young’s modulus were 30 GPa in the direction of the tubule and 15 GPa transversely to the tubule, with a Poisson’s ratio ranging from 0.3 to 0.4. This suggests that dentin is stiffer along the direction of the tubule.

Enamel mechanically works to crush food during chewing and protects dentin thanks to its wear resistance. The elasticity of the underlying dentin prevents enamel from fracturing easily. However, because of its ectodermal origin, enamel does not possess vessels and cells; therefore, it cannot repair or grow once it has been secreted and matured. Hence, crack propagation due to oblique loads may cause chipping and damage in this layer. Therefore, when a replacement material for enamel is to be selected, the focus should be on its hardness and ability to absorb shocks. In recent years, many restorative materials, such as plastic (acrylic), metal, and porcelain, have been developed for dentistry applications, even though many patients prefer ceramic crowns because of their excellent biocompatibility, esthetics, and chemical durability [12].Their use has diffused since the 1990s, in spite of the fact that porcelain is a brittle material characterized by a very high risk of breakage. In order to solve this problem, porcelain was fused with metal, which prevented the formation of stress cracks [13]. Zirconia was later introduced to replace ceramics, due to its remarkable mechanical strength [14,15,16]. The excellent properties of zirconia derive from the phase variations occurring during heating and compaction processes. In fact, zirconia has three crystalline phases: a monoclinic phase at room temperature, which, at 1000 °C, turns into a tetragonal phase and, then, becomes stable above 2000 °C thanks to the addition of yttrium (YSZ) or magnesium (MSZ). This fundamental step was designed to preserve the crystalline structure when zirconia cools after being sintered at high temperatures. The tetragonal phase offers a greater mechanical strength than the monoclinic phase. In dentistry, yttrium-Stabilized zirconia (YSZ) is the most common formulation for the fabrication of dental restorations, such as crowns or bridges, due to its high strength (800–1200 MPa) and its ability to maintain its shape and size over time. In addition, during the cooling of zirconia, there is a 3–4% volume expansion that retards crack propagation (the fracture toughness increases to 6–8 MPa [14]).

The evaluation of the strength characteristics is a fundamental step in assessing the mechanical behavior of dental restorations. In particular, numerical approaches developed for dentin usually rely on homogenization. A representative volume element is extracted from the dentin tissue and its mechanical characteristics are studied using a micromechanics-based approach. Alternatively, analytical models can be used to solve the micromechanical problem. One of the most efficient models was developed by Mori-Tanaka et al. [17], who supplied an empirical model accounting for tubular variation, geometry, and spatial variation in the tubules. This model allowed a clearer understanding of the non-uniform behavior of dentin [17,18].

Modern dentistry carefully considers the application of occlusal forces, stress distribution, and strains because these factors significantly influence the success of the restoration. In this regard, many experimental approaches as well as numerical techniques have been developed over the years to study the distribution of stresses in restorative elements under masticatory loads. In particular, the finite element method (FEM) has been shown to be a valid complement/alternative to the experimental assessment of the biomechanical behavior of restored teeth [19]. FEM solves an approximate problem defined by discretizing a geometric model describing the domain of the problem at hand into a 3D mesh comprising a finite number of elements of finite size and simple geometry. Elements are connected by characteristic points called nodes at which the structural response is computed. Each element of the FE model is subject to a set of applied loading conditions and kinematic constraints with the aim of deciding the global behavior of the discretized body.

Earlier studies published in the technical literature employed FEM to investigate the 2D biomechanical behavior of enamel, modeling this material as either isotropic or anisotropic [20] or purely isotropic [21]. Other studies later evaluated the biomechanical behavior of ceramic and zirconia restorative materials used for replacing natural enamel [22,23]. The present study aims to evaluate, using finite element analyses (FEA), the 3D biomechanical behavior of premolar teeth including restored crowns under axial and inclined occlusal loads. For that purpose, the stress distributions of restored teeth including ceramics or zirconia, are compared to those determined for natural teeth including isotropic enamel. The novelty of this study consists in the comparison between the two restorative materials and enamel, as all three materials are studied simultaneously. Additionally, the anisotropic behavior of enamel enhances accuracy. The bone region into which the premolar tooth is inserted is also modeled, and changes in stress distributions in the bone tissues are determined for the two restorative materials with respect to the case of natural teeth. The null hypothesis stating that varying crown material does not affect the results in terms of stress/deformation is not confirmed.

The rest of the article is structured as follows. Section 2 describes in detail the FE modeling process of the premolar tooth. Section 3 presents the FE solution options and the numerical results obtained for the different combinations of occlusal loads and materials. Section 4 discusses the results of Section 3 in the context of the technical literature. Section 5 summarizes the main findings and highlights the limitations and directions of future investigations.

## 2. Materials and Methods

### 2.1. CAD Model

**Figure 2:**
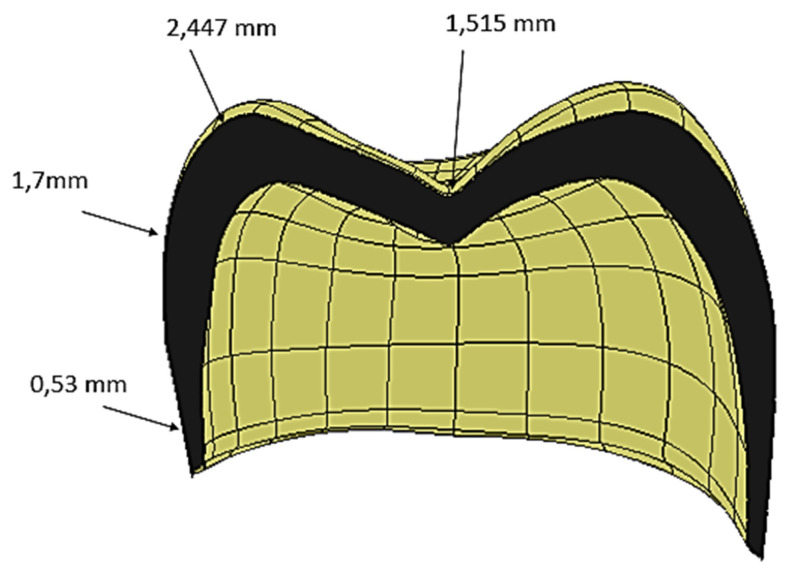
Cross-sectional view of the crown, showing the spatial variations in the crown thickness.

The computer-aided design (CAD) model was obtained from computed tomography (CT) scans of real premolar teeth, also considering the modeling conducted by Yoon et al. [24]. Recomposition and layering were processed using the Autodesk Inventor 2023^®^CAD (2023, San Francisco, CA 94105, USA) environment. The scan file coded in the .STL format was later converted into the .STP format. The thickness of the crown was selected from the recently published literature [24], where the thickness of the enamel layer was indicated to range from 0.3 mm to 2.5 mm (see Figure 2).

**Figure 3:**
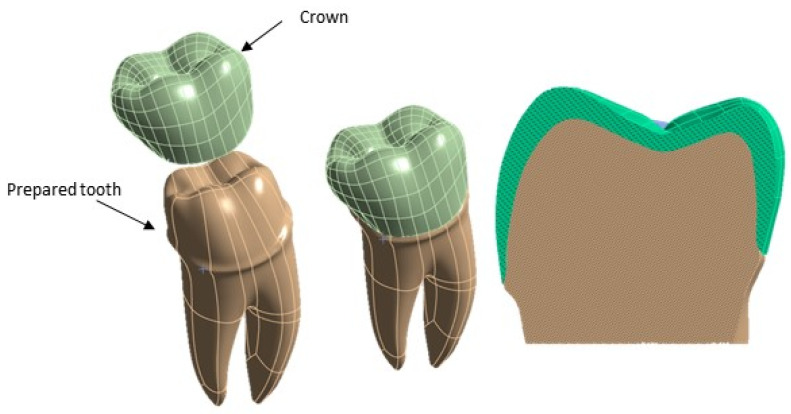
Components of the 3D model of the premolar tooth to be analyzed using FEA.

Subsequently, a 3D model of the premolar tooth suitable for accommodating the crown was prepared, which was then placed on the dentin [25,26], as it is shown in Figure 3.

**Figure 4:**
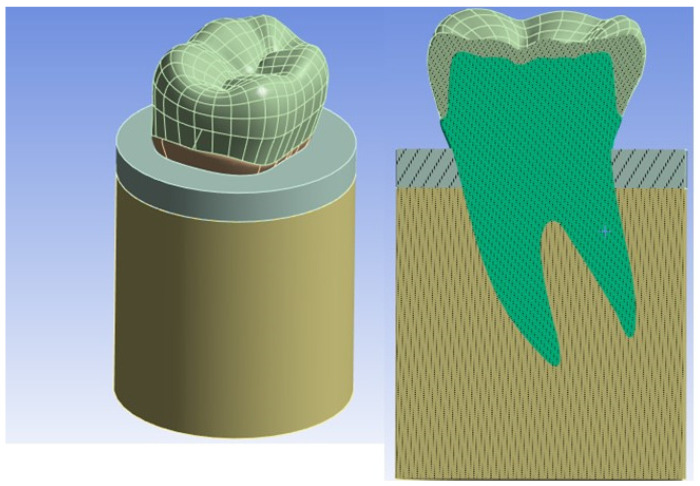
Overall model of the tooth inserted into the bone.

In order to make the FE model of the premolar tooth more reliable, a cylinder was created that simulated bone (cortical and trabecular) with a diameter of 15 mm and a height of 20 mm with a cortical bone thickness of 2 mm [27,28] (see Figure 4).

### 2.2. Finite Element Modeling

The CAD file of the 3D model of the premolar tooth was exported in the STP format and then input into the ANSYS Workbench 2023^®^ FEA software (R23, Canonsburg, PA, USA). This model was mainly discretized with tetrahedral elements (SOLID 187). In order to find the best trade-off between the accuracy of the results and the computational cost of FEA, a mesh convergence analysis was conducted by selecting the maximum principal stress as the target quantity. The element size was progressively reduced until the last three values of principal stress differed by less than 1%.

**Figure 5:**
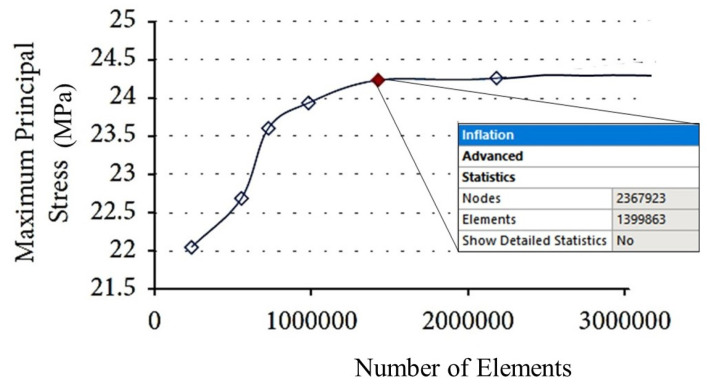
Results of the mesh sensitivity analysis.

**Figure 6:**
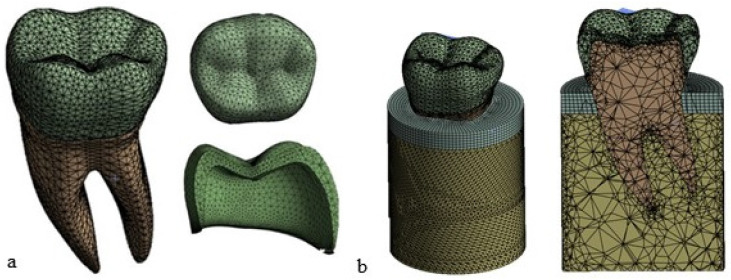
Mesh view display: (**a**) tooth and crown; (**b**) complete FE model of the tooth with the trabecular bone in yellow and the cortical bone in gray (element size = 0.8 mm).

Figure 5 shows that the maximum principal stress tends to reach an asymptotic value over a plateau starting at the red point denoted by about 140,000 elements. An element size of 0.8 mm was, therefore, chosen in this study for the finite element analyses. The details of the final meshes of the different parts of the FE model of the premolar tooth are presented in Figure 6.

### 2.3. Material Properties

The shaped tooth can be divided into three regions: crown, cement layer, and dentin. Three materials were selected in this study for the crown: enamel (natural tooth), ceramic, and zirconia (restored tooth). As for the bone, it can be divided into cortical bone and cancellous bone. Their difference is mainly due to their mechanical characteristics. In particular, the cortical bone is denser and has a better mechanical behavior than the trabecular bone. The presence of internal trabeculae leads to a reduced density and, hence, lower mechanical characteristics in the case of the trabecular bone [29,30]. To obtain information on the bone mechanical properties (i.e., Young’s modulus and Poisson’s ratio) and consequently on the deformation behavior, one can rely on mathematical relationships correlating the mass density of CT-scanned elements characterized by values expressed in Hounsfield units. These values can be related to the density of the element using Equation (1) [31].

(1) ρ=0.007764HU−0.05614

Wirtz et al. [32] proposed a mathematical relationship between the modulus of elasticity and density ofthe cortical and trabecular bones. That is:

(2) $$Ecort=2.065\times \rho^{3.09}$$

(3) $$Etrab=1.904\times \rho^{1.64}$$

The method described in [32] assumes that the bone material has an isotropic behavior with the same thermo-mechanical characteristics in all directions. However, other studies [33,34,35] proved that the bone response to external loads is best described by an anisotropic behavior (i.e., the thermo-mechanical characteristics are different in all directions) due to the non-homogeneity of the material because of the presence of trabeculae and the different responses to tensile and compressive loads. In view of this, the bone tissues were also modeled as anisotropic materials in this study using the mechanical properties listed in Table 1 [36].

As mentioned in the Introduction, dentin has an anisotropic behavior due to the microscopic nature of the tissue, which is formed by a set of tubules that confer anisotropy along the longitudinal directions of the tubules themselves. Table 2 lists the anisotropic properties of dentin input into the finite element model [37].

Munari et al. [38] compared the isotropic and anisotropic mechanical behaviors of natural enamel, finding marginal differences between the results obtained for these two hypotheses. Table 3 lists the modulus of elasticity E and the Poisson’s ratio values (the same values in all directions) used in this study [39].

Ceramic and zirconia restorative materials were also modeled as isotropic materials in this study on the basis of the data reported in the technical literature [40,41]. The corresponding mechanical properties used for the FEA are listed in Table 4.

Table 5 shows the strength limits of the materials studied in this paper. The listed values were extracted from [42,43,44,45].

### 2.4. Loads and Constraints

**Figure 7:**
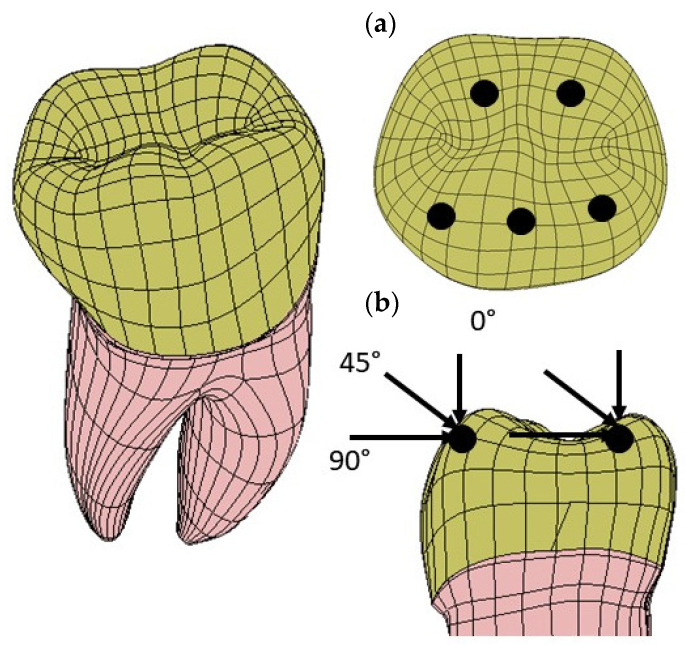
Load application points and the corresponding loading directions of the mastication forces simulating the maximum bite force: (**a**) three points are located on the inclined external sides of each buccal cusp and two points are located on the inclined internal sides of each lingual cusp; (**b**) there are three different load directions: vertical (axial), oblique (45°), and horizontal.

Three loading directions were considered to simulate mastication forces: a vertical (axial) load, an angled (45°) load, and a horizontal load. The loads were applied at five points on the occlusal surface of the dentin. These points simulated the possible points of contact during chewing. The intensity of the resultant load applied to the tooth was 280 N [40]. Figure 7 summarizes the applied forces and where they act on the tooth structure.

### 2.5. Kinematic Constraint Conditions

**Figure 8:**
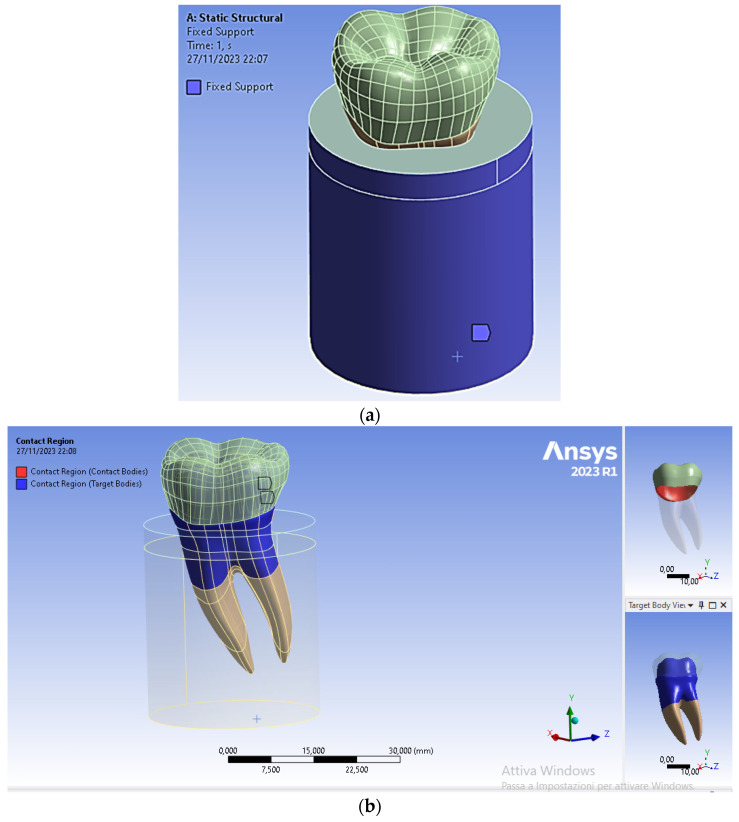
Boundary and contact conditions selected in the FEA of the restored premolar tooth: (**a**) fixed surfaces to simulate the rigid support provided by the bone region to the tooth; (**b**) type of contact between dentin and crown.

The lateral surfaces and the lower surface of the cylinder simulating the presence of the bone regions were fixed in all directions. To simulate a perfect osseointegration between tooth and bone, a fixed contact condition was selected (see Figure 8a). In addition, a fixed frictionless contact condition was also selected for the crown/dentin interface (see Figure 8b).

## 3. Results

The distributions of Von Mises equivalent stress and the corresponding stress peaks were the main output quantity obtained from FEA. The Von Mises stress supplies a single measure of the equivalent stress that, if exceeded, may yield the initiation of plastic deformation in the material. Stress distributions were plotted using ANSYS in the fashion of 3D maps with colors ranging from blue (low stress) to red (high stress).This allowed high stress regions to be promptly identified and, hence, to understand the mechanical behavior of the restored teeth under the occlusal loads shown in Figure 6 (i.e., 280 N resultant force acting in the apical direction, lingual, and 45° inclined direction applied to the occlusal surface of the crown).

**Figure 9:**
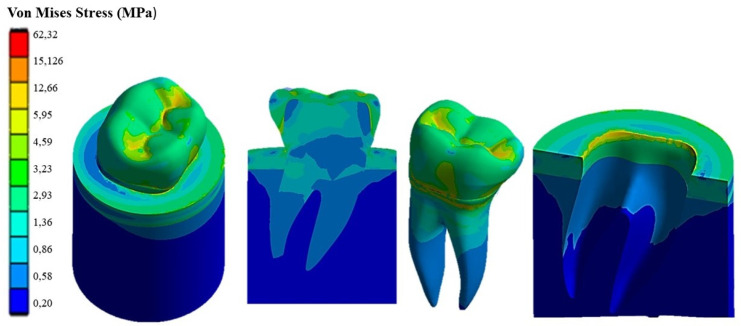
Von Mises stress distribution computed using FEA for a natural tooth (enamel crown) subject to an axial occlusal force.

**Figure 10:**
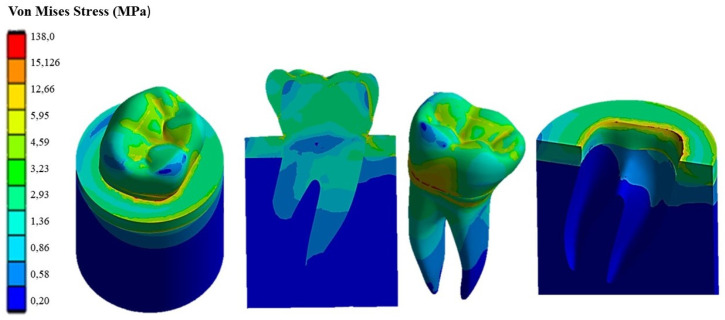
Von Mises stress distribution computed using FEA for a natural tooth (enamel crown) subject to occlusal forces inclined by 45°.

**Figure 11:**
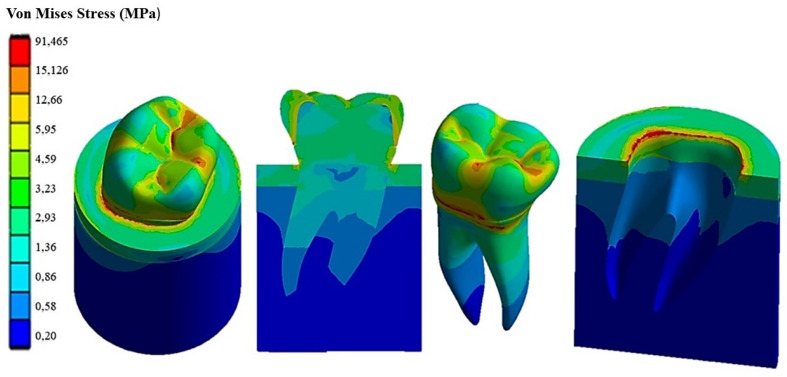
Von Mises stress distribution computed using FEA for a natural tooth (enamel crown) subject to a horizontal occlusal force.

Figure 9 shows the Von Mises stress distribution computed using FEA for the natural tooth subject to a 280 N occlusal load acting in the vertical direction. Figure 10 and Figure 11, respectively, show the Von Mises stress distributions obtained using ANSYS for the 45° inclined load and the horizontal load acting on the premolar tooth.

It can be seen that applying the load to the tooth in the apical direction (Figure 9) leads to a maximum Von Mises stress of 62.32 MPa in the cervical part of the tooth. Stresses in the occlusal surface range from 3 to 15 MPa with localized peaks in the grooves. Furthermore, looking at Figure 9, it can be seen that the occlusal load is equally distributed between two roots, which are stressed in the same way by a stress of about 3 MPa, with a concentration of stress in the area corresponding to the cervical part of the crown.

Figure 10 shows how the 45° inclined load generates load components in the direction perpendicular to the apical direction as well. This causes Von Mises stress to increase up to about 138 MPa in the cervical area. However, the stress distribution becomes asymmetric at the tooth roots. The right root that is in the direction of the inclined occlusal force is more stressed, reaching a maximum stress of about 4 MPa vs. only about 3.2 MPa localized in the left root. Figure 11 demonstrates that the application of a load parallel to the occlusal surface generates a maximum equivalent stress of about 91.465 MPa. However, unlike the other two load configurations previously analyzed, in this case, the occlusal surface of the crown is more stressed: a 58.32 MPa stress peak vs. only 12 MPa (vertical load) and 15 MPa (45° inclined load).

As expected, the cortical bone region had a higher stress than the spongy bone region in all load configurations because of its higher stiffness. This generated a phenomenon of stress shielding in the trabecular bone. The highest stress at the tooth–bone interface was observed for the inclined loading configuration: about 76 MPa vs. only 46 MPa computed for the axial load configuration and only 53 MPa computed for the horizontal load.

**Figure 12:**
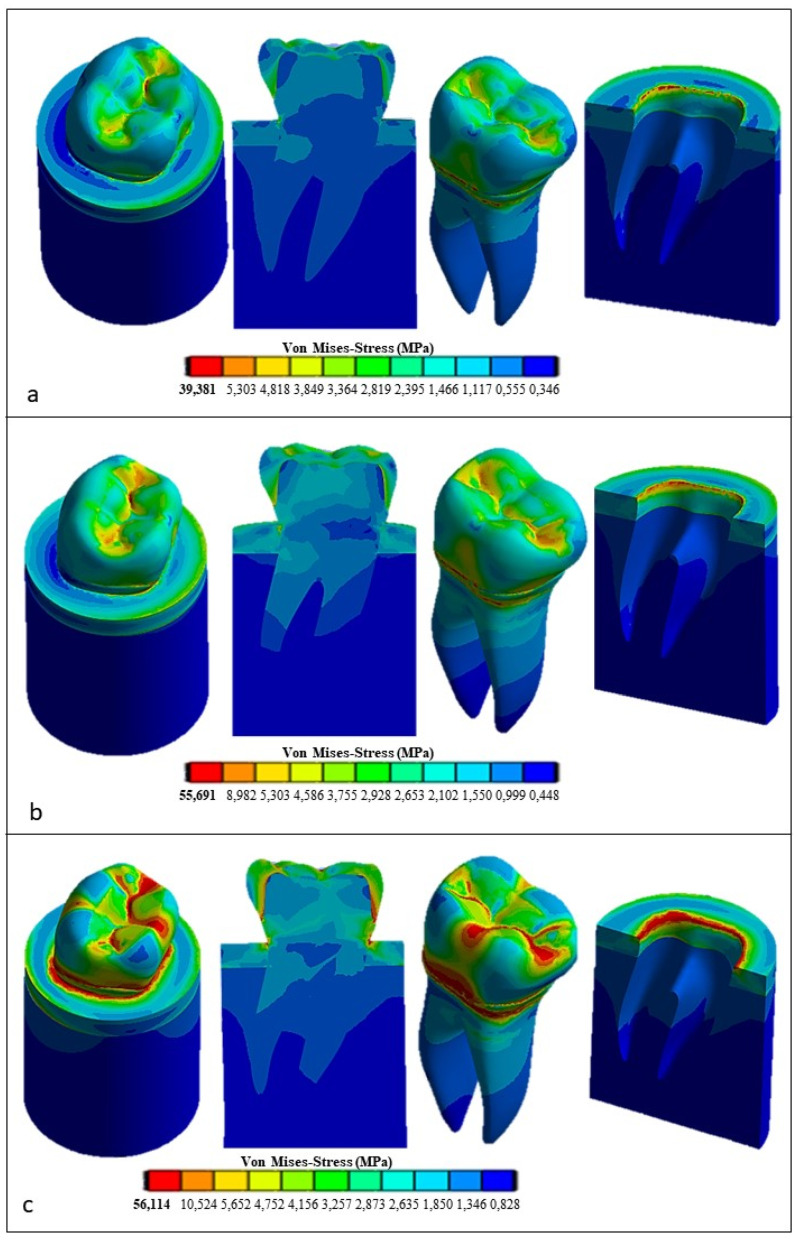
Von Mises stress distributions computed using FEA for the restored tooth with a ceramic crown: (**a**) vertical load, (**b**) 45° inclined load, and (**c**) horizontal load.

Figure 12 presents the Von Mises stress distribution computed using ANSYS for the restored tooth including ceramic as a replacement material for the crown. In the case of axial load (see Figure 12a), the maximum stress developed in the model is lower than that determined for the natural tooth (i.e., only 39.381 MPa vs. 62.32 MPa) and tends to be homogeneously distributed over the entire occlusal surface. At the roots, the stress is similar to its counterpart determined for the natural tooth (about 18 MPa). By inclining the force to 45° (see Figure 12b), the equivalent stress increased over the whole tooth structure in an analogous way to the case of the natural tooth with an enamel crown, yet it remained considerably lower: only about 55.691 MPa vs. about 138 MPa, respectively. Finally, the horizontal load (see Figure 12c) resulted in only a slight increase in the stress with respect to the case of the 45° inclined load, only 56.114 MPa vs. 55.691 MPa, respectively, while in the case of the natural tooth, the maximum stress varied toa large extent, dropping from about 138 MPa to about 91.465 MPa.

**Figure 13:**
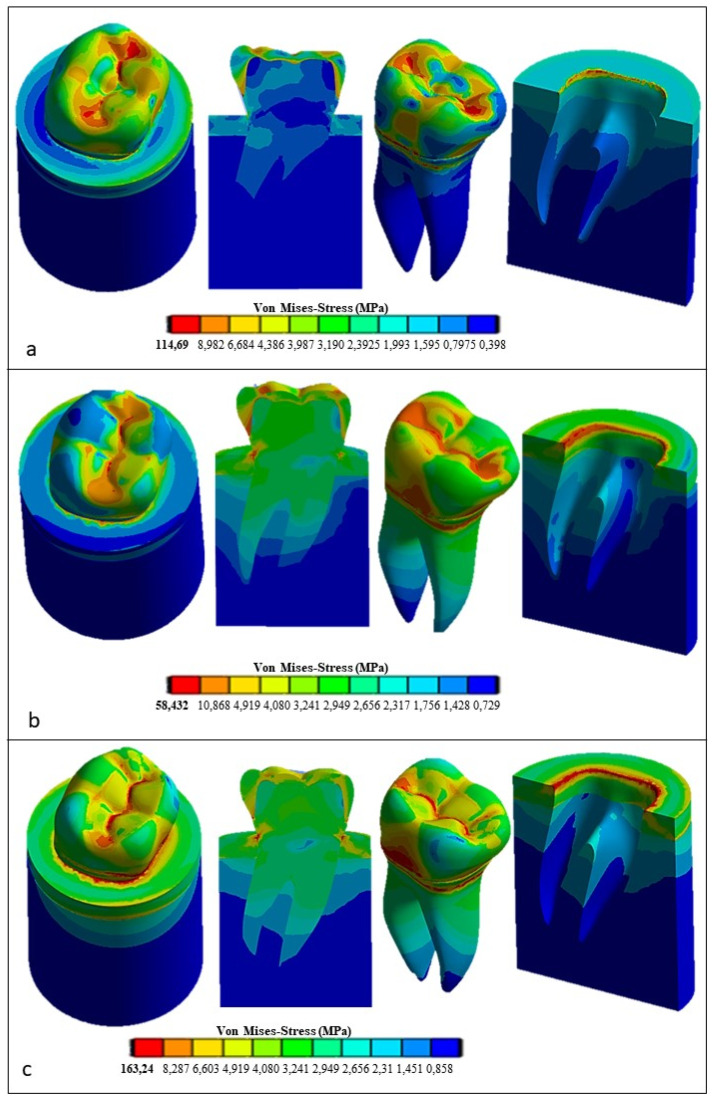
Von Mises stress distributions computed using FEA for the restored tooth with a zirconia crown: (**a**) vertical load; (**b**) 45° inclined load; and (**c**) horizontal load.

**Figure 14:**
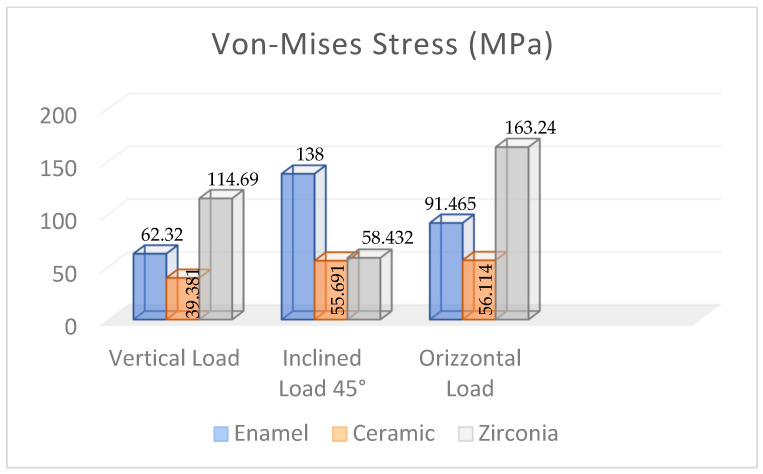
Maximum Von Mises stress values computed using FEA for the different dental structures under various loading conditions.

Figure 13 presents the FEA results obtained for the restored premolar with a zirconia crown. In the case of axial and horizontal loadings, the computed stresses in the tooth structure were significantly higher than those computed for both the restored premolar tooth with a ceramic crown and the natural tooth with an enamel crown. The stress values computed for the occlusal load inclined at 45° were similar for both restored teeth. Figure 14 summarizes the main results obtained in terms of the maximum stress whose values are reported in the graph for the different combinations of tooth structure (i.e., natural, restored with a ceramic crown, and restored with a zirconia crown) and loading conditions (axial, 45° inclined, and horizontal forces). All stress values computed using ANSYS were lower than the strength limits indicated in Table 5. Figure 14 shows that using the ceramic crown restoration allows us to obtain a similar mechanical behavior under all loading conditions experienced by the tooth structure (i.e., axial, inclined, and horizontal forces). Conversely, the zirconia-crown-restored tooth shows significant stress peaks for perfectly axial and horizontal loads. In terms of the stress level and the consequent risk of fracture, the ceramic crown restoration may require more attention to avoid physical damage, while the zirconia crown restoration may be more tolerant to mechanical stresses. This is confirmed in Figure 12: in the case of the ceramic restoration, the maximum stresses are localized at certain critical points and such a non-uniform distribution may increase the probability of initiating fracture phenomena.

## 4. Discussion

The present study investigated the effect of the dental crown material (enamel for the natural tooth and ceramic and zirconia crowns for the restored teeth) on the 3D stress distribution of a mandibular premolar tooth. Three-dimensional finite element models, including the tooth structure and a cylindrical block consisting of the cortical bone and trabecular bone to support the tooth, were developed for this study. The zirconia restoration was selected because its mechanical properties provide excellent mechanical strength in dental applications. These characteristics can be greatly improved by firing at a high temperature, which triggers transformation hardening that is opposed to the propagation of cracks [46]. Therefore, the zirconia restoration has significantly higher mechanical properties than other restorative materials, like ceramics [47,48]. In [49], it was shown that zirconia is stable at 1170 °C, but it has a cubic structure at 2370 °C [50]. Most zirconia-based prosthetic structures are made of yttrium-stabilized zirconium polycrystals (3Y-TZP) [48]. The most important advantage of this stabilizer is its high fracture toughness and flexural strength [51]. Despite the popularity of zirconia, various complications have been reported in the technical literature for this restorative material [52,53,54]. For example, there can be residual thermal stresses due to mismatches between the coefficient of thermal expansion and differences in the modulus of elasticity between zirconia and the coating material, which may facilitate a chipping fracture [55,56].

Other clinical trial data [57,58,59] focused on the fact that the type of fixation agent between the crown and abutment can affect the retention of zirconium-based crowns and the durability of the implant. Furthermore, the thickness of the cement layer significantly influences crown retention. If the cement layer is thin, the gap between the prosthetic crown and abutment may become very small, thus increasing the forces needed to extract the prosthetic crown from the abutment and compromising the durability of the prosthetic application [58]. Ceramics are brittle materials and, hence, very susceptible to a risk of fractures [53]. To reduce this risk, the ceramic is melted with metal alloys that provide a certain toughness to the structure. Aceramic crown, compared to the zirconia one, provides a very natural and translucent appearance, similar to that of the natural tooth. Even though it is esthetically better, ceramic is less resistant to fracture than zirconia [60]. In addition, even when fitting with a natural tooth, the ceramic crown does not require a removal of the tooth structure that is as considerable as that with zirconia, as the ceramic is to be thinner.

One approach to investigating the integrity of dental structures is to use destructive tests that apply load cycles on the tooth element through a ball or bar [61]. This in vitro method has limitations because the testing machine setup may not directly simulate the actual oral conditions resulting from variations in masticatory load as well as the directionality of the load. Sorrentino et al. [62] evaluated in vitro the mechanical strength of a monolithic zirconia crown obtaining about 1655 N vs. only about 1400 N for ceramics. This confirmed the higher mechanical strength of zirconia in the case of masticatory loads. FEA was used to mimic the intraoral conditions to evaluate the fracture strength of various materials used in dental restorations [63,64,65,66]. Alsadon et al. [51] obtained similar results for zirconium-coated crowns and zirconium–porcelain-composite-coated crowns: the peak stresses were, respectively, equal to63.6 MPa and 50.9 MPa. Fathy et al. [67] studied the finite element stress distribution in fully milled or layered zirconia crowns. They found that the single-material zirconia-restored crown was stiffer than the layered crown restoration. Other FEA studies [67,68,69] also investigated stress distribution in bone based on the selected material for the crown: the porcelain coating led to a reduction in stresses in the bone due to the lower elastic modulus compared to zirconia.

The results obtained in present paper are consistent with those reviewed above. In fact, stiffness variations in the restorative material used for replacing enamel may result in a significant stress reduction in the tooth elements. Such an effect is more pronounced if the horizontal components of the occlusal loads predominate, thus stressing the occlusal surface. Angular and horizontal loads cause the stress distribution to become wider than in the case of the vertical load. These loads are generated more in the cervical region than in the apical region of the tooth.

Some studies [70] evaluating cement spaces demonstrated how the presence of a larger cement layer localizes peak stresses in marginal areas of the concrete, making it more susceptible to failure. In this study, the cement layer was omitted to study precisely the critical condition in which there is a higher contact stress between the crown and the prosthetic system. In addition, it was seen that, compared to natural enamel, the ceramic crown generated less stress in the bone than the zirconia crown. A prosthetic restoration with an osseointegrated implant in the bone is currently being developed also considering that stresses in the bone that are very low may cause bone resorption. Therefore, zirconia turns out to be the best replacement material of natural enamel in terms of optimizing osteointegration at the tooth–bone interface.

A limitation of the present study is in the modeling of the contact at the tooth–bone interface. In this paper, the modeling of periodontal ligament (PDL) was omitted because several studies [71,72,73], focusing on the influence that the periodontal ligament can have on the stress transfer from teeth to the bone, indicated such influence to become significant only if the study focuses exclusively on the tooth–bone contact. Conversely, if the research scope is to investigate the overall mechanical behavior of the tooth structure, the presence of the periodontal ligament can be omitted. Moreover, since the mechanical behavior of the periodontal ligament is not yet fully understood, it is very difficult to reliably model it in the context of FEA. Controversial studies [74,75] indicated a hyperplastic behavior is more suited than a viscoelastic behavior for the periodontal ligament. Other studies [76,77] stated that varying the PDL’s constitutive behavior may change the position of the tension peaks, which can be translated from the cervical area in the case of the PDL’s hyperelastic behavior to the distal area if a viscoelastic or elastoplastic behavior is assumed for the periodontal ligament. The relative limitations of this study mainly involve the simplification of the model, as it was not possible to model all components, such as the periodontal ligament, due to the limited availability of studies allowing the PDL’s mechanical characterization. Additionally, the dependence on input data, such as loads and constraints, may have led to less accurate results. The mechanical properties of the materials used in the simulation significantly influence the outcomes. There are studies considering isotropic or anisotropic bone, and different behaviors may lead to the creation of anomalous stress fields at the bone–tooth interface. Therefore, FEA studies should be compared with in vitro tests to ensure result accuracy.

## 5. Conclusions

In this study, FEA confirmed itself as an extremely useful tool for evaluating stresses in a complex biomechanical structure comprising different materials, such as a restored mandibular premolar tooth. Using 3D modeling and numerical analyses, it was possible to understand how the selection of the crown restorative material affects the stress distribution with respect to the natural tooth. Despite having excellent esthetic characteristics, ceramic has a lower resistance to occlusal loads than zirconia. It was seen that varying the stiffness of the selected crown replacement materials (i.e., zirconia and ceramics) significantly affected the stress distribution in the restored tooth. Occlusal load direction also affected the intensity and distribution of the transmitted stress. In particular, the application of a horizontal load significantly increased the stress on the occlusal surface of the zirconia crown with respect to the ceramic crown restoration, which appeared to be rather insensitive to the direction of applied force. The effect of the cement layer between the crown and dentin was not considered because the focus was more on the analysis of the occlusal surface of the tooth. The stress at the tooth–bone interface was also influenced by the presence of the periodontal ligament, which, due to the general analysis of transmitted stress, was not considered in this study. Future investigations should include the correct implementation of these elements in order to more accurately assess the transmitted stresses through the tooth structure interfaces.

## Figures and Tables

**Table 1:** Anisotropic mechanical properties of the bone tissues input into the FEA of the restored teeth.

Material	E*_z_* (GPa)	E*_y_* (GPa)	E*_x_* (GPa)	$\nu_{xy}$	$\nu_{yx}$	$\nu_{xz}$	$G_{xy}\left(\mathrm{GPa}\right)$	$G_{yz}\left(\mathrm{GPa}\right)$	$G_{xz}\left(\mathrm{GPa}\right)$
Cortical bone	17.9	12.5	26.6	0.28	0.18	0.31	7.1	4.5	5.3
Cancellous bone	0.21	1.148	1.148	0.055	0.322	0.055	0.068	0.434	0.068

**Table 2:** Anisotropic mechanical properties of dentin input into the FEA of the restored teeth.

Material	E*_z_* (GPa)	E*_y_* (GPa)	E*_x_* (GPa)	$\nu_{xy}$	$\nu_{yx}$	$\nu_{xz}$	$G_{xy}\left(\mathrm{GPa}\right)$	$G_{yz}\;\left(\mathrm{GPa}\right)$	$G_{xz}\left(\mathrm{GPa}\right)$
Dentin	17.07	5.61	5.61	0.30	0.33	0.30	1.7	6	1.7

**Table 3:** Isotropic mechanical properties of natural enamel input into the FEA of the restored teeth.

Material	E (GPa)	ν
Enamel	72.7	0.30

**Table 4:** Crown materials properties.

Material	E (GPa)	ν
Zirconia	205	0.22
Porcelain	68.9	0.28

**Table 5:** Tensile strength and compressive strength of the analyzed materials.

Material	Tensile Strength (MPa)	Compressive Strength (MPa)
Enamel	11.5	384.0
Dentin	105.5	297.0
Zirconia	745.0	2000.0
Ceramic		330
Cortical bone	135	205
Trabecular bone		10.44

## Data Availability

All experimental data to support the findings of this study are available from the corresponding authors upon request.
